# Reconfigurable Architecture for Multi-lead ECG Signal Compression with High-frequency Noise Reduction

**DOI:** 10.1038/s41598-019-53460-3

**Published:** 2019-11-21

**Authors:** Mehdi Hasan Chowdhury, Ray C. C. Cheung

**Affiliations:** 1 Department of EE, City University of Hong Kong, Kowloon, Hong Kong; 2 Department of EEE, Chittagong University of Engineering & Technology, Chittagong, Bangladesh

**Keywords:** Biomedical engineering, Electrical and electronic engineering

## Abstract

Electrocardiogram (ECG) is a record of the heart’s electrical activity over a specified period, and it is the most popular noninvasive diagnostic test to identify several cardiac diseases. It is an integral part of a typical eHealth system, where the ECG signals are often needed to be compressed for long term data recording and remote transmission. Reconfigurable architecture offers high-speed parallel computation unit, particularly the Field Programmable Gate Array (FPGA) along with adaptable software features. Hence, this type of design is suitable for multi-channel signal processing units like ECGs, which usually require precise real-time computation. This paper presents a reconfigurable signal processing unit which is implemented in ZedBoard- a development board for Xilinx Zynq −7000 SoC. The compression algorithm is based on Fast Fourier Transformation. The implemented system can work in real-time and achieve a maximum 90% compression rate without any significant signal distortion (i.e., less than 9% normalized percentage of root-mean-square deviation). This compression rate is 5% higher than the state-of-the-art hardware implementation. Additionally, this algorithm has an inherent capability of high-frequency noise reduction, which makes it unique in this field. The confirmatory analysis is done using six databases from the PhysioNet databank to compare and validate the effectiveness of the proposed system.

## Introduction

The integrated use of information technology and electronic communication in the health sector is recognized as an eHealth system^1^. It is a technical platform that comprises diagnostic tools and microcomputer units. eHealth is especially a vital resource for the health care of the people from remote regions and developing countries as it helps the patients to consult specialist physicians using telecommunications. Even in developed countries, electronic health records are being kept for follow-up treatment. Therefore, electronic communication and data recording are the fundamental system components of an eHealth system.

Cardiovascular disease (CVD) is a generic term to indicate the disorders related to heart and blood vessels. According to the World Health Organization (WHO) CVDs are responsible for 31% of all worldwide deaths, taking approximately 18 million lives per year^2^. Regular monitoring of a patient’s heart condition is the primary requirement for diagnosing the underlying cardiac diseases. A record of the electrical potential of heart muscle depolarization over a specified time is called the Electrocardiogram (ECG or EKG), and this is measured by placing multiple electrode pads (sensors^3–5^) on specified places of the chest. These electrodes are connected to an ECG machine for the signal processing and recording. The electrocardiogram is the most popular noninvasive diagnostic test to determine cardiac health^6^. It had been used as a diagnostic test in clinical medicine for more than 70 years^7^. At present, ECG is one of the routine tests for any complete medical evaluation, and it is an indispensable part of a modern eHealth system^8^. It should be noted that, while recording ECG, the measurement is done by determining the potential difference between predefined locations on the body surface. This generates distinct ECG vectors known as the ‘leads’. Common clinical standard uses a 12-lead ECG system for assessing a patient’s cardiac health^9^.

As a part of an eHealth system, ECG signal requires to be transmitted to a distant location for telediagnosis and stored for future treatment reference. Thus the necessity of ECG signal compression arises. Compression helps to reduce the required bandwidth for data transmission. It also saves space for storing the ECG data. This paper presents the design and implementation of an FPGA based reconfigurable system for ECG compression.

Various noises affect ECG signal during the data accusation^10^ and transmission process^11^. The high-frequency noise is one of the primary reasons for ECG signal distortion^12^, and it mostly contains the powerline interference (50–60 Hz) and the electromyographic (EMG) noise (100–500 Hz)^13^. One of the added benefits of the proposed system is its built-in capability of high-frequency noise cancellation. Consequently, additional high-frequency noise filtering mechanism in the eHealth system will be redundant and the overall system complexity will be reduced.

The FPGA is essentially an integrated circuit (IC) which can be reconfigured for any assigned task, hence the name ‘reconfigurable computing’^14^. It is widely used for ASIC (Application Specific Integrated Circuit) prototyping and hardware verification^15^. Because of the unique capability of parallel computation^16^, FPGAs are significantly faster in the applications where simultaneous computation is needed for multiple processes^17^. In a typical electrophysiological monitoring unit, data accusation is often made by collecting signals from multiple channels. As mentioned earlier, a clinical ECG has a 12 lead standard. This means while processing the data it needs 12 concurrent signal processing. Therefore, FPGA is used as the computation unit in the proposed architecture.

The fundamental focus of an effective ECG compression is efficient transmission and data storage without losing critical diagnostic information. Many methods are found in the literature for the compression of the ECG signal. Based on the techniques, they can be classified as follows: (i) time domain compression, (ii) transform domain compression, (iii) compression by parameter extraction, and (iv) hybrid method. The original time-domain ECG signal features are scrutinized, and redundant data points are discarded in the time domain compression method^18^. As no significant data conversion or transformation is needed in this method, this scheme offers relatively fast processing^19^. The second compression method is the transform domain compression, and it operates by converting the original signal to frequency^20^ or spatiotemporal^21^ signal. In this method, the signal is processed and compressed after the transformation. Consequently, it takes longer processing time than the first method, though this method is capable of more efficient compression. Compression by parameter extraction is the third ECG compression method. This scheme requires a complex feature extraction from the given signal. Various learning methods are applied to determine the parameter characteristics, which are then used to compress the original signal and conserved thereafter for decompression^8,22^. Some contemporary feature extraction techniques include supervised dictionary learning^23^, object detection^24,25^, background information retrieval^26,27^, and deep learning^28^. Hybrid compression method combines the particulars from time-domain, transform-domain, and parameter extraction methods to create an efficient compression scheme^29,30^. The last two methods involve more computation than the first two methods. Hence, they require more processing time and resources.

Nonetheless, most of the above researches are mainly concentrated on the software level^31,32^. This paper is more focused on the system level implementation of a real-time ECG compression algorithm. Therefore, a fast signal processing method with low hardware resource requirement is perceived while conceiving the appropriate ECG compression algorithm. The proposed system employs a transform domain compression method based on Fast Fourier Transformation (FFT) technique. This system is hardware implemented and demonstrates a significant improvement in compression efficiency without any vital signal deformation. Moreover, it has a built-in high-frequency noise cancellation ability, which makes it unique from the other implemented systems. A comparative study of the proposed system with the other methods is presented in the “Result Analysis” section.

## Methodology

The Fast Fourier Transform is a highly optimized form of the Discrete Fourier Transform (DFT), which takes a sequence of sampled data in the time domain and computes the frequency component of that data sequence. DFT is defined by the folllowing equation^33^:

1 $$X(k)=\mathop{\sum }\limits_{n=0}^{N-1}\,x(n){e}^{-j2\pi kn/N},\,k=0,1,2,\ldots ,N-1$$

Here, each of the single frequency components, $X(k)$ is calculated by considering each of the time domain samples, $x(n)$. Hence, ${N}^{2}$ addition and multiplication is needed to compute $N$ number of samples. To recover the time domain components from frequency samples IDFT (Inverse DFT) is used, and it is defined^33^ by:

2 $$x(n)=\frac{1}{N}\mathop{\sum }\limits_{k=0}^{N-1}\,X(k){e}^{j2\pi kn/N},\,n=0,1,2,\ldots ,N-1$$

Again, Eq. (2) requires ${N}^{2}$ addition and multiplication to process $N$ number of samples in spatio-temporal domain. This large number of mathematical operation often creates a computational burden. Hence, FFT and IFFT (Inverse FFT) algorithms are developed^34^. FFT reduces the calculation complexity of DFT by factorizing its matrix into sparse arrays in which the majority of the elements are zero^35^. For N number of points the required number of complex multiplication in the DFT is ${N}^{2}$, whereas in the similar case, only $(N\mathrm{/2)}lo{g}_{2}N$ complex multiplication is needed in the FFT algorithm^33^. As an example, if 128 number of point is considered, DFT and FFT involve 16,384 and 448 complex multiplication respectively. Thus, FFT improves the computational speed by a factor of 36.6 in this case^34^.

Using the FPGA as the signal processing hardware gives the unique capability to decompose the complete multichannel architecture into a series of structurally identical single channel core units.

**Figure 1:**
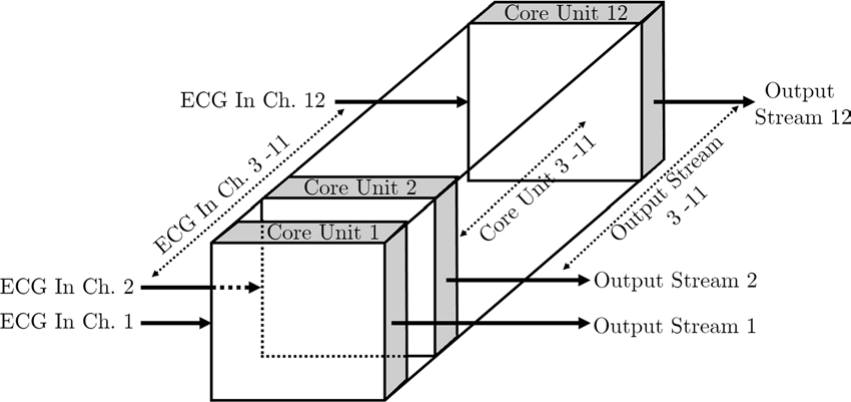
Core units.

**Figure 2:**
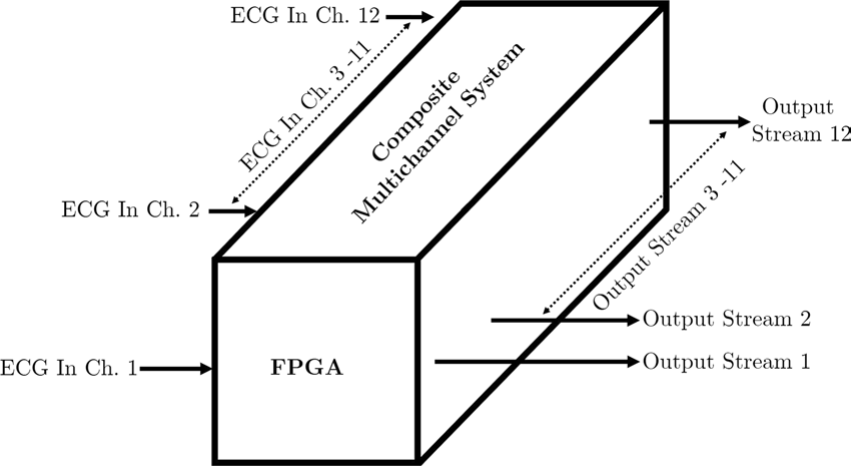
Complete multichannel system architecture.

Consequently, a composition of these elementary segments becomes the complete multichannel system as shown in Figs. 1 and 2. The concept of parallel computation in the FPGA can also be understood from this figure. Twelve signals from the ECG signal accusation unit are fed into the FPGA based signal processing core, and these signals are simultaneously processed to produce the desired real-time outputs.

**Figure 3:**
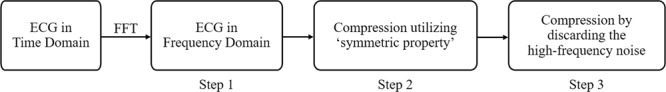
Compression Steps.

The proposed compression algorithm works in three steps. At first, the original time domain ECG signal is subjected to FFT for transforming it into the frequency domain. Next, the first level compression is done utilizing the ‘symmetric property’ of this frequency response. Finally, additional compression is performed by discarding the high-frequency noise components. Figure 3 demonstrates a simple block diagram to clarify the compression steps.

**Figure 4:**
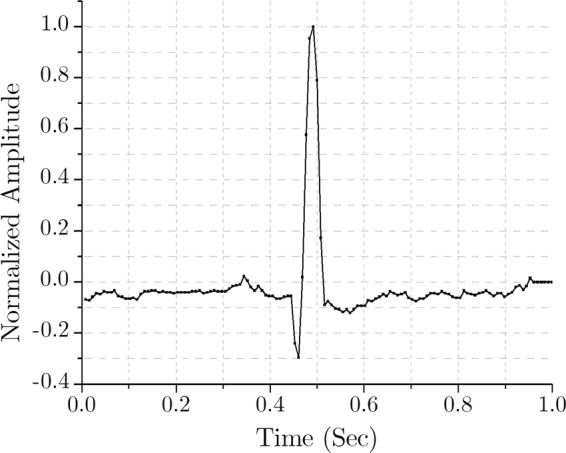
Time-domain ECG of a normal sinus rhythm.

A bit accurate MATLAB program is developed, and a standard ECG signal from the MIT-BIH Normal Sinus Rhythm Database (collected from the PhysioBank^36^ record: 16265, sampling frequency 128 Hz) is used to demonstrate the proposed method. As mentioned earlier, for simplicity, this primary model is designed for a single channel ECG compression and decompression. The complete multichannel scheme is implemented in hardware and described in the section ‘FPGA Implementation’. Figure 4 shows an epoch of a single channel signal from the aforementioned ECG, displaying a time window of 1 second.

**Figure 5:**
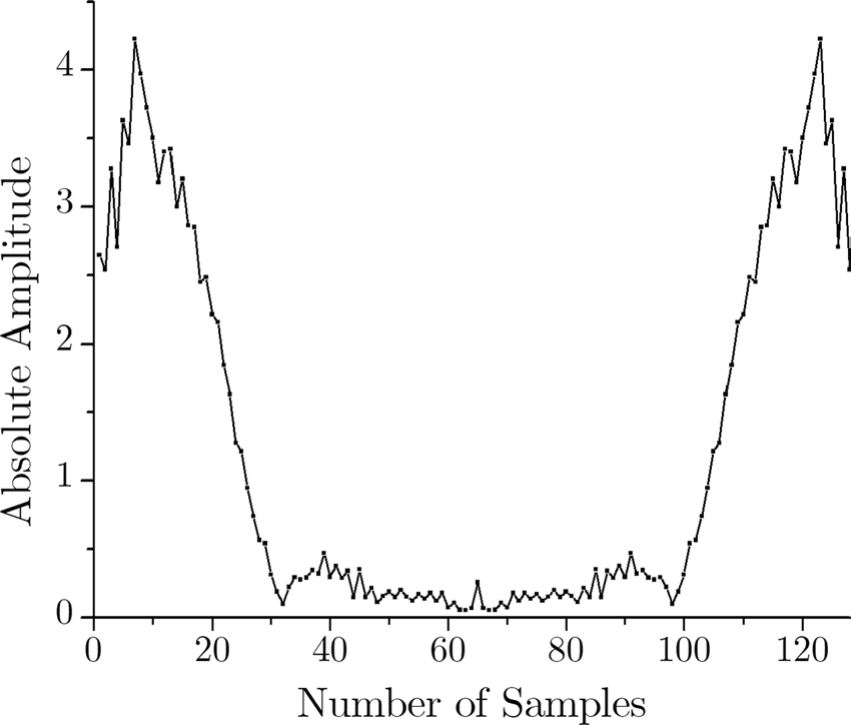
Frequency-domain (128-point FFT) output.

As mentioned earlier, the sampling frequency is 128 Hz. Therefore, this epoch of 1 second corresponds to 128 data samples in the time domain. As the first step of compression, FFT is used to transform this time domain signal to the frequency domain. Here, 128-point FFT is applied to the signal of Fig. 4, and the outcome is 128 complex samples in the frequency domain. This frequency response in absolute value is shown in Fig. 5.

It is mathematically established that applying FFT to a real signal results in a symmetric complex signal (with the real and imaginary parts)^37^. Since the input ECG is a real signal, the FFT output is a complex one, and as expected, it is symmetric in nature. The data in frequency domain sample number 65 through 128 are redundant to store in the memory because they can be regenerated by simply mirroring the rest of the data. Therefore, the first level of compression is performed by discarding half of the FFT data.

**Figure 6:**
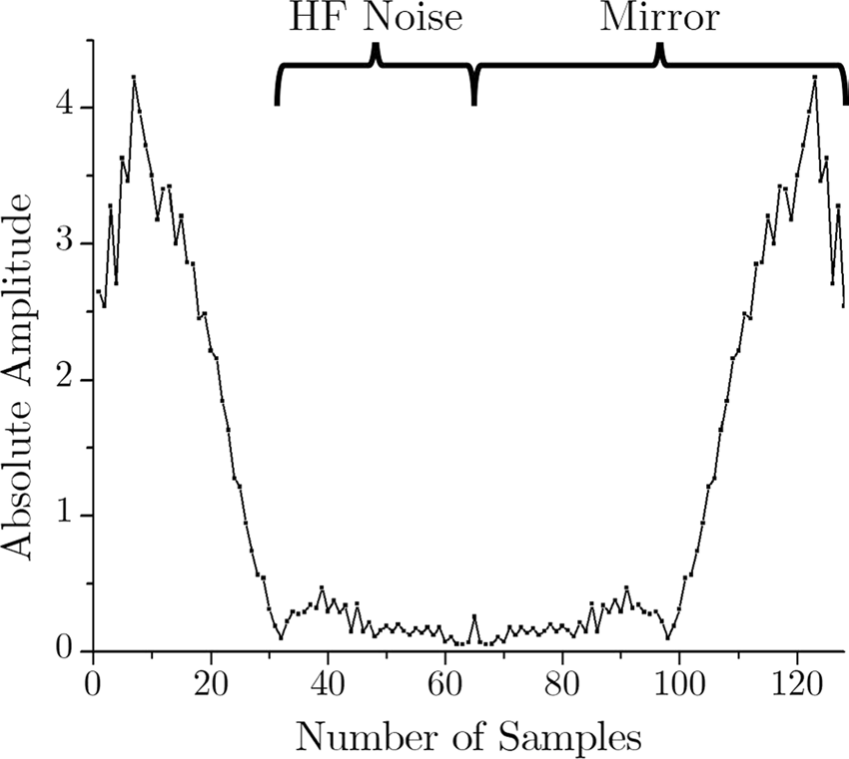
FFT output of the ECG signal.

**Figure 7:**
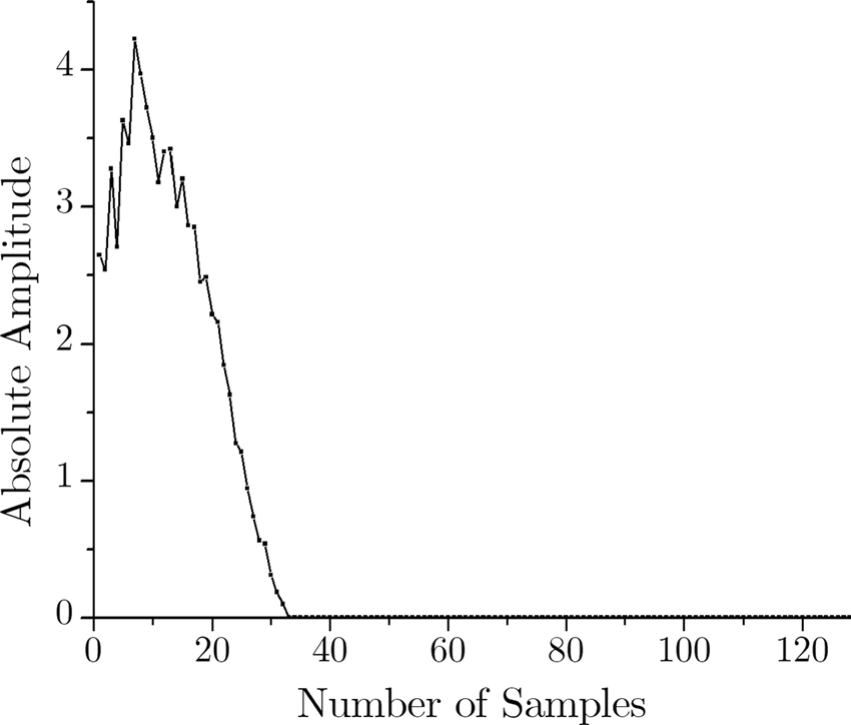
Compressed ECG in frequency domain.

At this point, it should be noted that the ECG frequency components are dominant in the lower frequency range (<30 Hz)^38^. Here, the sampling frequency is 128 Hz. Nyquist theorem dictates that the input signal can represent up to half of its sampling frequency^33^. Hence, the input signal can have frequency components up to 64 Hz. In this case, 128-point FFT is applied; hence it has a 0–64 Hz band, which is conveniently represented by the respective sample numbers. Therefore, for the second level of compression, data of the sample number 33 through 64 are dropped, as they are essentially the high-frequency (33–64 Hz) noise components. Thus the essential lower frequency components (0–32 Hz) prevail while the high-frequency (HF) noises (>32 Hz) are discarded in the compressed signal. These two-level compression are shown in Figs. 6 and 7 respectively.

To explain the compression ratio, let us take the above example in consideration. Here, the input time domain signal has 128 real number. At first, consider this numbers in an input bin A. The FFT output is 128 complex numbers. Putting the real and imaginary number in the separate bins will result in 2 bins B and C with 128 number in each of them, totaling 256 numbers in the memory. This means the resultant memory size is, in fact, taking double space compared to the input. However, after the first level of compression, the size of the bin B and C will be half, as these bins will now contain 64 numbers each. At this point the total output memory contains 128 numbers, which is the same as the input bin. Finally, the second level of compression is executed by dropping the high-frequency noise signals. This process will further size down bin B and C. Now, these bins hold 32 numbers each. Therefore, the final output bins will contain 64 numbers in total. The compression ratio (CR) is defined by^39^,

3 $$CR( \% )=\frac{size\,of\,the\,input\,stream-size\,of\,the\,output\,stream}{size\,of\,the\,input\,stream}\times 100$$

For this particular example, the size of the input and output stream is 128 and 64 respectively. In this case, the compression ratio is 50%. However, CR can be easily adjusted by modifying how much high-frequency components is to be discarded.

**Figure 8:**
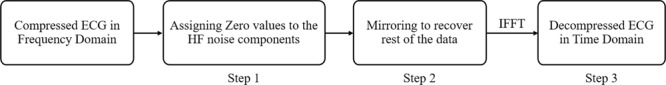
Decompression Steps.

**Figure 9:**
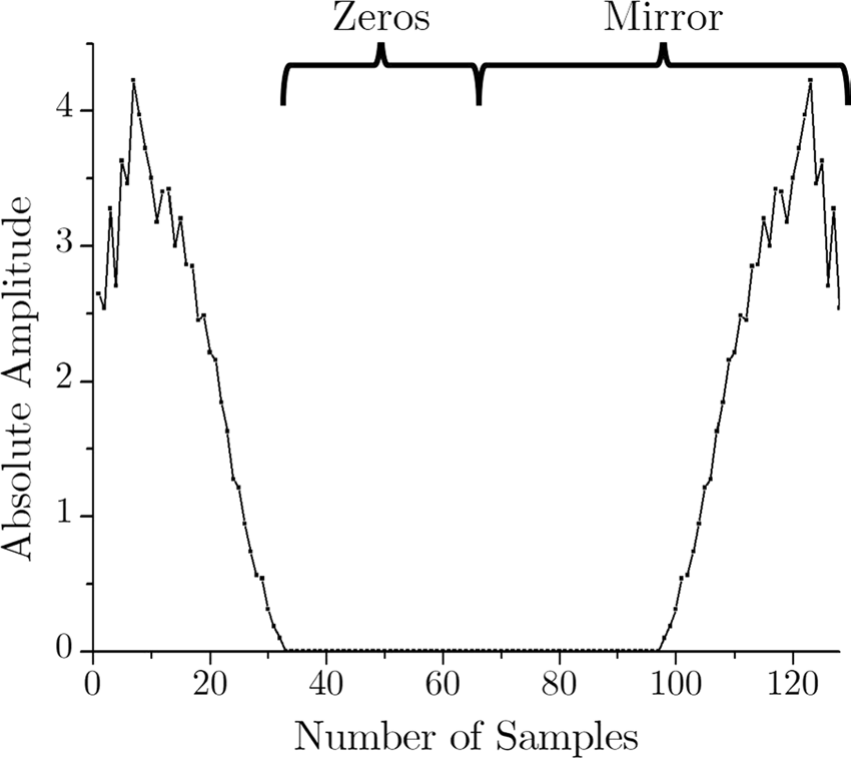
ECG decompression in frequency domain.

**Figure 10:**
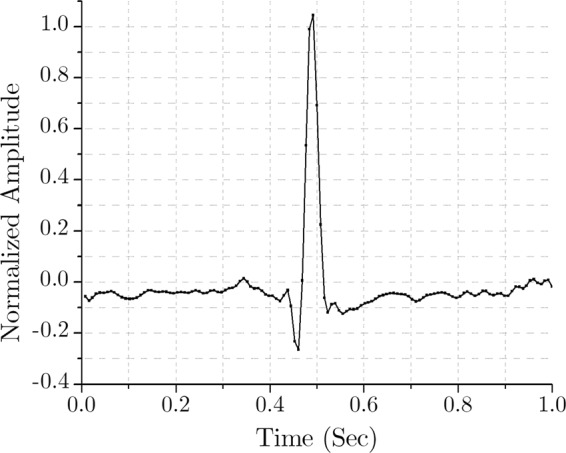
IFFT to retrieve the temporal ECG.

The decompression algorithm is somewhat a reverse technique of the compression method. Figure 8 demonstrates a simplified block diagram of the decompression process. Taking the compressed ECG signal from the preceding example, the main objective of decompression is to recover the data of the frequency sample number 33 through 128. As mentioned earlier, the discarded data of the sample number 33 through 64 are actually high-frequency noise components. Therefore, as the first step of decompression, the data corresponding the sample number 33 through 64 are substituted by zeros to ensure the HF noise reduction. The second step is mirroring the frequency components of sample number 1 through 64 to regenerate the data of the sample number 65 through 128. Finally, the frequency domain decompressed signal is transformed into the time domain using the IFFT. Figures 9 and 10 show the decompression process and the restored time-domain ECG signal.

## FPGA Implementation

ZedBoard is an optimum cost-effective development board manufactured by Digilent. This board employs Xilinx Artix-7 FPGAs coupled with a dual-core ARM Cortex-A9 processor, which is specially optimized for digital signal processing (DSP) applications^40^. ZedBoard is used as the processing unit for implementing the proposed scheme in hardware level.

**Figure 11:**
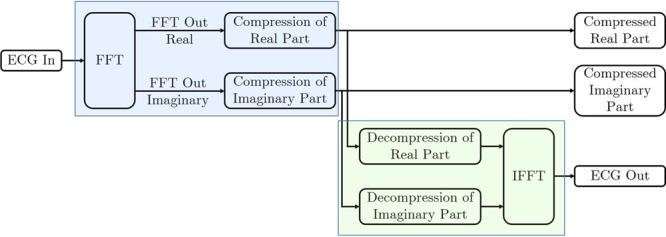
Block diagram of the System Generator unit.

**Figure 12:**
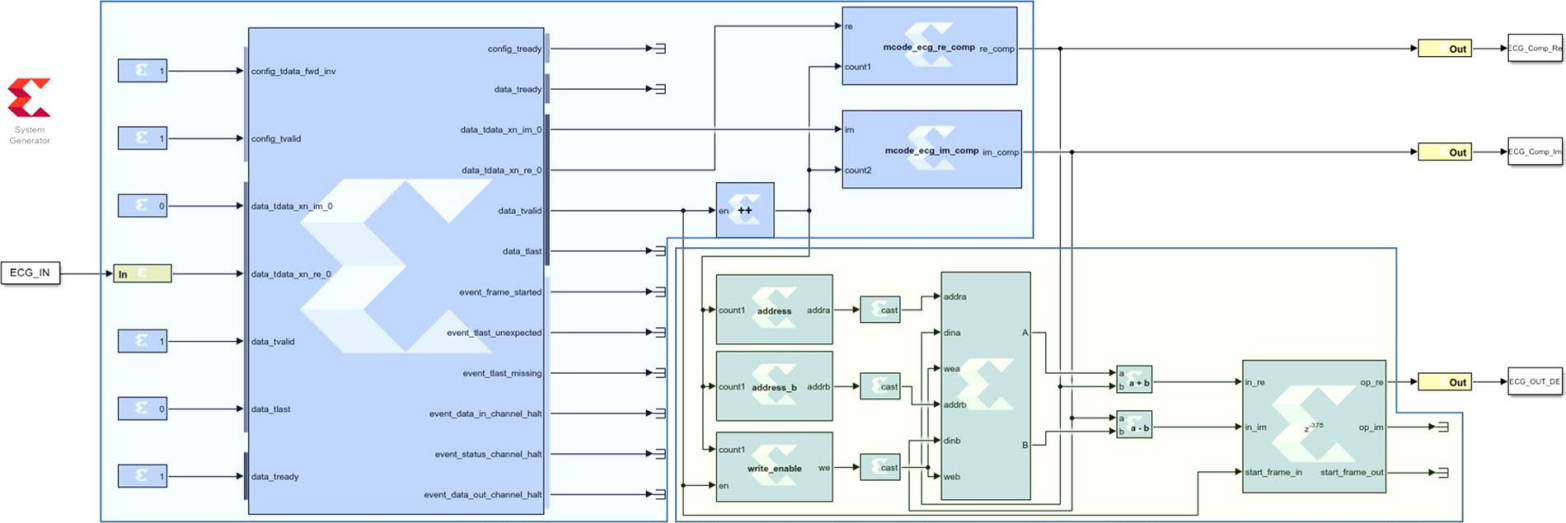
Screenshot of the practical System Generator unit.

System Generator is one of the design tools for the implementation of DSP algorithms in Xilinx devices, and it is used to program the ZedBorad in this research. System Generator works as a toolbox for Simulink, a graphical programming environment of MATLAB. A simple diagram of the practical hardware design is shown in Fig. 11 and the screenshot of the practical system generator design is illustrated in Fig. 12.

It should be noted that this core processing unit consists of both compression (blue shaded portion of the Figs. 11 and 12) and decompression (green shaded portion of the Figs. 11 and 12) unit. If necessary, the practical arrangement in the eHealth system can be designed either as a compression unit (for transmission system) or as a decompression unit (for reception system).

**Figure 13:**
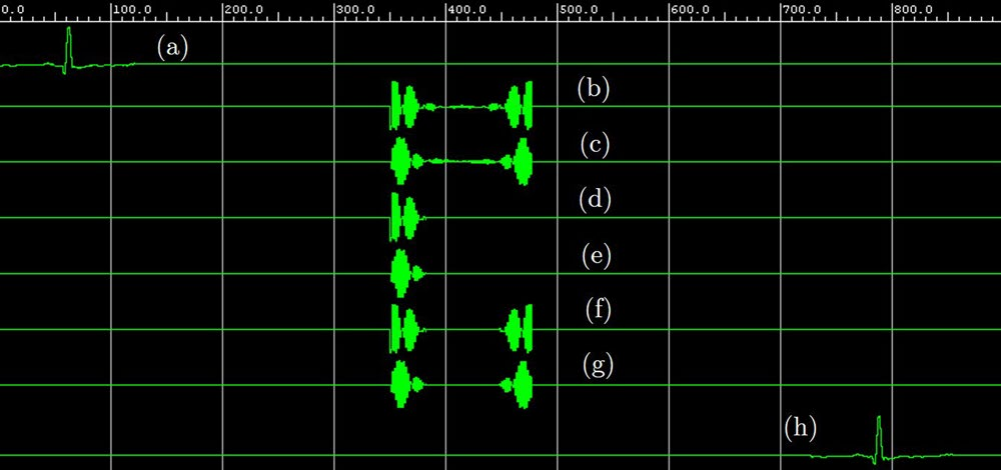
Different signals in the Xilinx Waveform Viewer: (**a**) ECG input, (**b**) real part of the ECG in frequency-domain, (**c**) imaginary part of the ECG in frequency-domain, (**d**) compressed real part of the ECG in frequency-domain, (**e**) compressed imaginary part of the ECG in frequency-domain, (**f**) decompressed real part of the ECG in frequency-domain, (**g**) decompressed imaginary part of the ECG in frequency-domain, (**h**) decompressed FPGA ECG output in time-domain.

The compression unit is composed of three subunits— one FFT module and two compression subunits. The input ECG signal is fed into the FFT unit, and it returns two FFT outputs as real and imaginary numbers. These real and imaginary parts are compressed in the following units. The output of these compression units are the desired compressed signal which can be stored or transmitted for farther use. In the design, these compressed signals are used as the input of the decompression unit. Here, the decompression unit is also comprised of three subunits. Two of them are for decompressing the compressed frequency-domain ECG signal. The final subunit is the IFFT module, which converts the decompressed frequency-domain ECG signal to the desired time-domain ECG as the output. Figure 13 shows signals of different processing stages in the Xilinx Waveform Viewer.

Table 1 shows the hardware resources required in the implementation of a single channel ECG processing unit (i.e. the core unit) on Zedboard. This ECG processing unit comprises both compression and decompression subsystems. Comparing the number of used resources with the available resources on Zedboard, it is possible to identify the plausible maximum number of core unit implementation on a single Zedboard. Here, the core unit utilizes 34% of the available DSPs, featuring the largest percentage of available resource consumption. Hence, two ECG processing units (including both compression and decompression subsystems) can be implemented on one Zedboard. Consequently, six Zedboards will be required for the implementation of a twelve channel system with compression-decompression capability. However, for an eHealth transmission or recording system, only the capability of compression is adequate. In this case, a twelve channel ECG compression unit can be built using only one Zedboard. To built an eHealth reception or restoring system, only decompression ability is required. Therefore, four Zedboards will be needed to design a twelve channel ECG reconstruction unit.

**Table 1:** System resource utilization.

Resource ↓	Unit				
	Compression	Decompression	Common	Total	Available
BRAMs	1	2	0	3	140
DSPs	9	66	0	75	220
LUTs	2480	3172	113	5765	53200
Registers	2915	4108	8	7031	106400

In this research, the prototype uses Zedboard for hardware implementation. This development board is a low-cost option with a relatively low-end FPGA as the processing unit. Though employing this board offers a budget development prospect, this is also one of the limitations of the implemented system. The high-end boards provide advanced FPGAs with more hardware resources. Therefore, the processing unit can accommodate more core units than the proposed prototype. Furthermore, complex algorithms can be adopted if adequate hardware resources become available. This constitutes a scope for future development.

## Result Analysis

As mentioned earlier, the proposed system compresses the ECG signal in the frequency domain. This compression is done in two vital steps: discarding the symmetric data and removing the high-frequency components. The reconstruction of the discarded symmetric data does not involve any data loss as it can be done by simple data mirroring. However, as the high-frequency components are considered as noises and permanently discarded during the compression process, the restored ECG signal does not inherit the high-frequency components. Though this process attributes as a low pass noise filter, the removal of high-frequency components makes the decompressed ECG somewhat different from the original signal.

**Figure 14:**
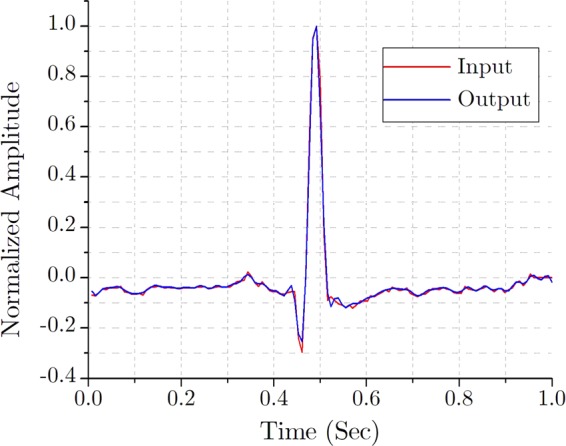
Superimposed input and output ECG traces.

For a preliminary graphical comparison, the raw input and the recovered output ECG are superimposed in the Fig. 14. It can be readily comprehended from a thorough visual inspection that, these two ECG traces are practically indistinguishable. As the ECG compression should aim to maximize compression efficiency without the deterioration of the signal quality, both the compression efficiency and the signal quality are evaluated while analyzing the performance of the proposed system.

Compression efficiency indicates how much the processed data is compressed compared to the raw data. The most common parameter to evaluate the compression efficiency is to measure the “Compression Ratio (CR)”^39^. CR is defined by Eq. (3) in the “Methedology” section which can be rewritten in the form of Eq. (4) for further discussion.

4 $$CR( \% )=(1-\frac{size\,of\,the\,output\,stream}{size\,of\,the\,input\,stream})\times 100$$

It should be noted that in some literature, CR is also mentioned as the Data-volume Saving (DS) as it essentially indicates the percentage of data being saved by the compression^41^.

It goes without saying that the quality of the reconstructed ECG signal must be satisfactory to avoid misdiagnosis. There are several parameters to assess the quality of the decompressed ECG. Among them, the “Percentage Root mean square Difference (PRD)” is the most common and the “Normalized version of Percentage Root mean square Difference (PRDN)” is the most accurate^39^. PRD and PRDN are defined by the Eqs (5) and (6) respectively.

5 $$PRD( \% )=\sqrt{\frac{\mathop{\sum }\limits_{n=1}^{N}\,{[x(n)-\tilde{x}(n)]}^{2}}{\mathop{\sum }\limits_{n=1}^{N}\,{[x(n)]}^{2}}}\times 100$$

6 $$PRDN( \% )=\sqrt{\frac{\mathop{\sum }\limits_{n\mathrm{=1}}^{N}{[x(n)-\tilde{x}(n)]}^{2}}{\mathop{\sum }\limits_{n\mathrm{=1}}^{N}{[x(n)-\bar{x}(n)]}^{2}}}\times 100$$

Here, $x(n)$ is the original signal, $\tilde{x}(n)$ is the reconstructed signal and $\bar{x}(n)$ is the mean of the original signal. According to^39,42^ and^43^, if the value of PRDN is less than 9% then the quality of the reconstructed signals are considered as “very good”.

The acceptable ECG sampling frequency range for legitimate clinical use is 100–1000 Hz^44^. Therefore, to cover this frequency range, six databases are used to assess the performance of the proposed system. These datasets are downloaded from the PhysioBank^36^ and presented in the Table 2 with relevant references.

**Table 2:** Used databases for the performance evaluation.

Database Name	Sampling Frequency, *F*_*S*_	Reference
MIT-BIH NSR Database	128 Hz	Goldberger *et al*.^36^
European ST-T Database	250 Hz	Taddei *et al*.^46^
MIT-BIH Arrhythmia Database	360 Hz	Moody *et al*.^47^
MAC ECG Database	500 Hz	Behravan *et al*.^48^
ANSI/AAMI EC13 Test Waveforms	720 Hz	AAMI^49^
ECG DMMLD Database	1000 Hz	Johannesen *et al*.^50^

**Figure 15:**
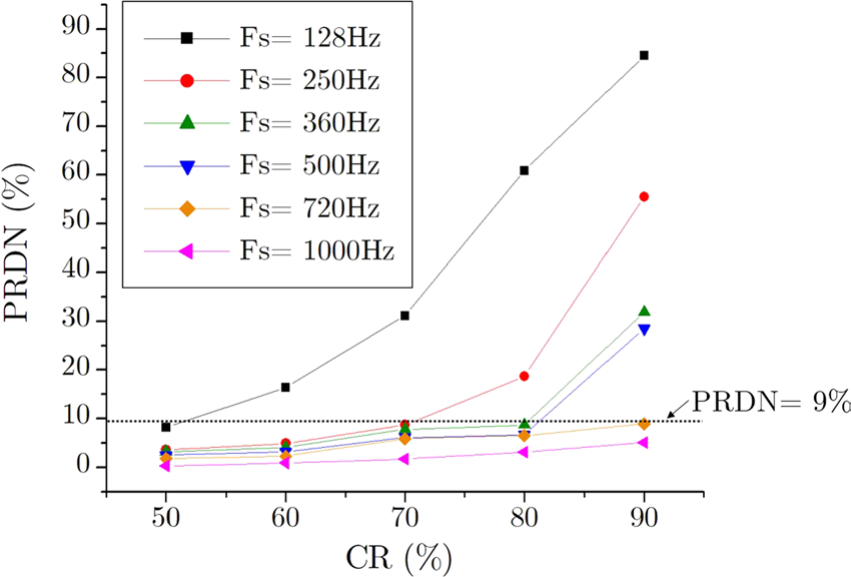
CR vs. PRDN for different sampling frequency.

The compression ratio of the processed ECG can be controlled by simply adjusting how much high-frequency components to be discarded. However, dropping too much data will, of course, distort the resultant signal from the original one. Figure 15 shows the relationship between the Compression Ratio and the Normalized Percentage Root mean square Difference for various sampling frequency.

**Figure 16:**
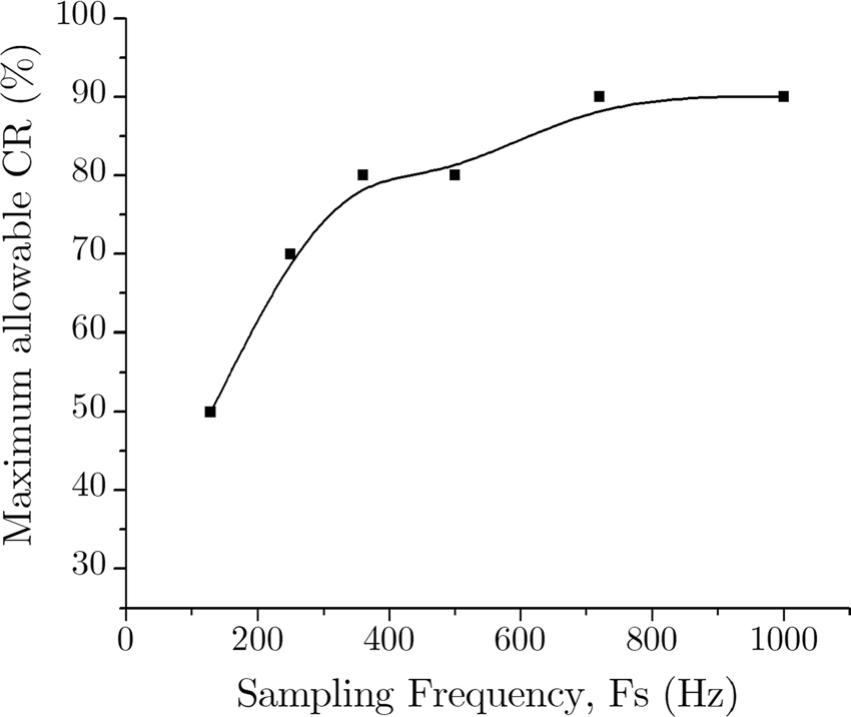
Maximum allowable CR (%) for different sampling frequency.

As expected, a higher percentage of CR gives a higher rate of PRDN. This case is more severe for the lower sampling frequency but much less prevailing for the higher one. This phenomenon can be explained by using the Nyquist theorem as mentioned in the “Methodology” section. The ECG signals with higher sampling frequency inherently possess greater high-frequency noise components in it. Hence, it is possible to discard much of these noisy data during the compression stage without declining the signal quality. Actually, this property is suitable for practical applications; as for similar signals, a lower sampling frequency already consumes fewer data. As the signals with higher sampling frequency occupy more data, it makes sense to compress it with greater compression efficiency. Figure 15 also indicates the 9% PRDN line, which is the boundary condition for being a “very good” compressed signal. Considering this 9% PRDN as the higher limit of signal distortion we can find out the maximum allowable compression ratio for different sampling frequency as shown in Fig. 16.

At this point, it should be noted that, though discarding high-frequency components distorts the compressed signal from the original one, it acts as a high-frequency noise reduction process. This is an added benefit of the proposed compression system. Using FPGAs for hardware implementation plays another vital role in this research. As a reconfigurable device, the same FPGA units can be easily configured for different compression ratio for different sampling frequency when required. This indeed makes the eHealth system more flexible for global implementation.

To conclude the result analysis a comparative study between the proposed system and the previous works is presented in Table 3. In spite of being the most critical quality measurement parameter, PRDN is not specified in some of the papers. However, PRD is measured for all cases. Therefore, PRD is also included as a quality measurement parameter for this study. For this table the performance of the proposed system is measured for 720 Hz sampling frequency using ANSI/AAMI EC13 Test database.

**Table 3:** Comparison of compression performance.

Reference	Method Used	CR (%)	PRD (%)	PRDN (%)	Hardware Implementation	Real-Time Execution	Noise Reduction
Cox *et al*.^18^	Time Domain	83	31.0	—	No	No	No
Abenstein *et al*.^19^	Time Domain	80	11.0	—	No	No	No
Alam *et al*.^21^	Transform Domain	90	5.1	6.5	No	No	No
Ziran *et al*.^20^	Transform Domain	95	10.6	—	No	No	No
Adamo *et al*.^51^	Parameter Extraction	42	1.1	17.3	No	No	No
Elgendi *et al*.^8^	Parameter Extraction	78	0.5	—	No	No	No
Elgendi *et al*.^22^	Parameter Extraction	83	1.9	—	No	No	No
Wang *et al*.^29^	Hybrid	94	7.3	—	No	No	No
Ma *et al*.^30^	Hybrid	94	1.7	25.7	No	No	No
Lee *et al*.^45^	Hybrid	95	2.9	40.6	No	No	No
Lai *et al*.^52^	Transform Domain	81	0.2	3.7	Yes	Yes	No
Luo *et al*.^53^	Transform Domain	85	0.2	2.6	Yes	Yes	No
Proposed	Transform Domain	90	1.5	8.8	Yes	Yes	Yes

A few of the previous works (and^20,29,30^,^45^) can compress the ECG at a higher rate than the proposed one. However, their signal quality is less than the new system, and these works are not implemented at the hardware level. Among the hardware implemented works, the proposed method has the highest compression ratio (at least 5% improvement) while maintaining the accepted PRDN limit. It is the only implemented system which can reduce noises from the ECG while compressing it. The overall signal quality is actually improved during the compression process as the high-frequency noise is reduced. This makes the new system unique. The higher data volume savings with better signal quality ensures economical telemedicine and electronic health record system. Therefore, the proposed system is more suitable for upper-level applications like eHealth systems.

## Conclusion

This paper presents a novel reconfigurable system architecture for electrocardiographic signal compression. To the best of our knowledge, this is the first hardware implemented ECG compression system which can reduce the embedded high-frequency noise from the original signal while compressing it. This unique feature enhances the overall ECG signal quality for further diagnosis. Because of its high compression performance and superior signal quality, this system is ideal for the incorporation with a contemporary eHealth care system. Furthermore, similar methods and system designs can be utilized for compressing other signals, especially the signals with distinct frequency dependence. The FPGA design can be used for prototyping a dedicated application specific integrated circuit in the future. This will make the system suitable for wearable wireless ECG monitoring systems.

## Data availability

The datasets analyzed during the current study are available in the PhysioBank repository, www.physionet.org/physiobank/.

## Supplementary information

LaTeX Supplementary File

## References

[1] Mitchell J (2000). Increasing the cost-effectiveness of telemedicine by embracing e-health. J. Telemedicine Telecare.

[2] World Health Organization (ed.) *Hearts: Technical package for cardiovascular disease management in primary health care* (World Health Organization: WHO, 2016).

[3] Wu W, Pirbhulal S, Zhang H, Mukhopadhyay SC (2019). Quantitative assessment for self-tracking of acute stress based on triangulation principle in a wearable sensor system. IEEE journal biomedical health informatics.

[4] Wu W, Zhang H, Pirbhulal S, Mukhopadhyay SC, Zhang Y-T (2015). Assessment of biofeedback training for emotion management through wearable textile physiological monitoring system. IEEE Sensors J..

[5] Pirbhulal S, Zhang H, Wu W, Mukhopadhyay SC, Zhang Y-T (2018). Heartbeats based biometric random binary sequences generation to secure wireless body sensor networks. IEEE Transactions on Biomed. Eng..

[6] Chowdhury, M. H. & Hossain, Q. D. Development of two wireless ECG monitoring systems and their performance assessment. In *International Conference on Informatics, Electronics Vision*, 459–464, 10.1109/ICIEV.2018.8641068 (2018).

[7] Shouleice R, Bass G (2002). From bench to bedside-developments in electrocardiology. The Eng. Journal, Inst. Eng. Irel..

[8] Elgendi, M., Mohamed, A. & Ward, R. Efficient ECG compression and QRS detection for e-health applications. *Sci. Reports* **7**, 10.1038/s41598-017-00540-x (2017). 10.1038/s41598-017-00540-x PMC5428727 28352071

[9] Chowdhury, M. H., Hossain, Q. D., Saha, P. & Rahaman, M. M. Design, fabrication and performance evaluation of a three electrode ECG recorder. In *International Conference on Innovations in Science, Engineering and Technology*, 10.1109/ICISET.2016.7856500 (2016).

[10] Poungponsri S, Yu X-H (2013). An adaptive filtering approach for electrocardiogram (ECG) signal noise reduction using neural networks. Neurocomputing.

[11] Chowdhury MH, Hossain QD, Hossain MA, Cheung RCC (2019). Single feed circularly polarized crescent-cut and extended corner square microstrip antennas for wireless biotelemetry. Int. J. Electr. Comput. Eng..

[12] Patro, K. K. & Kumar, P. R. De-noising of ECG raw signal by cascaded window based digital filters configuration. In *2015 IEEE Power, Communication and Information Technology Conference (PCITC)*, 120–124, 10.1109/PCITC.2015.7438145 (IEEE, 2015).

[13] Friesen GM (1990). A comparison of the noise sensitivity of nine QRS detection algorithms. IEEE Transactions on biomedical engineering.

[14] Li WX (2011). High-performance and scalable system architecture for the real-time estimation of generalized laguerrevolterra MIMO model from neural population spiking activity. IEEE J. on Emerg. Sel. Top. Circuits Syst..

[15] Chu, P. P. *FPGA Prototyping by Verilog Examples: Xilinx Spartan-3 Version* (John Wiley & Sons, Incorporated, Hoboken, 2008).

[16] Li WXY, Cheung RCC, Chan RHM, Dong Song TW, Berger TW (2013). Real-Time Prediction of Neuronal Population Spiking Activity Using FPGA. Biomed. Circuits Syst. IEEE Transactions on.

[17] Mano, M. M. *Digital design: with an introduction to the Verilog HDL*, 5^th^ ed., international ed. edn (Pearson Education Ltd., Harlow, Essex, 2013).

[18] Cox J. R., Nolle F. M., Fozzard H. A., Oliver G. C. (1968). AZTEC, a Preprocessing Program for Real-Time ECG Rhythm Analysis. IEEE Transactions on Biomedical Engineering.

[19] Abenstein John P., Tompkins Willis J. (1982). A New Data-Reduction Algorithm for Real-Time ECG Analysis. IEEE Transactions on Biomedical Engineering.

[20] Ziran P, Guojun W, Jiang H, Shuangwu M (2017). Research and improvement of ECG compression algorithm based on EZW. Comput. methods programs biomedicine.

[21] Alam, M. S. & Rahim, N. M. S. Compression of ECG signal based on its deviation from a reference signal using discrete cosine transform. In *2008 International Conference on Electrical and Computer Engineering*, 53–58, 10.1109/ICECE.2008.4769172 (IEEE, 2008).

[22] Elgendi M, Al-Ali A, Mohamed A, Ward R (2018). Improving remote health monitoring: A low-complexity ECG compression approach. Diagn..

[23] Zhao S (2015). Supervised dictionary learning for inferring concurrent brain networks. IEEE Transactions on Med. Imaging.

[24] Han J, Zhang D, Cheng G, Guo L, Ren J (2015). Object detection in optical remote sensing images based on weakly supervised learning and high-level feature learning. IEEE Transactions on Geosci. Remote. Sens..

[25] Zhang D, Han J, Li C, Wang J, Li X (2016). Detection of co-salient objects by looking deep and wide. Int. J. Comput. Vis..

[26] Han J (2013). Representing and retrieving video shots in human-centric brain imaging space. IEEE Transactions on Image Process..

[27] Cheng G, Zhou P, Han J (2016). Learning rotation-invariant convolutional neural networks for object detection in vhr optical remote sensing images. IEEE Transactions on Geosci. Remote. Sens..

[28] Han J (2015). Background prior-based salient object detection via deep reconstruction residual. IEEE Transactions on Circuits Syst. for Video Technol..

[29] Wang X, Chen Z, Luo J, Meng J, Xu Y (2016). ECG compression based on combining of EMD and wavelet transform. Electron. Lett..

[30] Ma J, Zhang T, Dong M (2015). A novel ECG data compression method using adaptive fourier decomposition with security guarantee in e-health applications. IEEE journal biomedical health informatics.

[31] Wang Y (2019). Multi-objective workflow scheduling with deep-q-network-based multi-agent reinforcement learning. IEEE Access.

[32] Wu W, Pirbhulal S, Sangaiah AK, Mukhopadhyay SC, Li G (2018). Optimization of signal quality over comfortability of textile electrodes for ecg monitoring in fog computing based medical applications. Futur. generation computer systems.

[33] Proakis, J. G. & Manolakis, D. G. *Digital signal processing*, 4^th^ edition edn. (Pearson/Prentice Hall, Upper Saddle River, NJ, 2007).

[34] Parker, M. *Digital signal processing 101*, 2nd edition edn. (Elsevier/Newnes, Amsterdam; Boston, 2017).

[35] Van Loan, C. *Computational Frameworks for the Fast Fourier Transform* (Society for Industrial and Applied Mathematics, 1992).

[36] Goldberger AL (2000). PhysioBank, PhysioToolkit, and PhysioNet: Components of a new research resource for complex physiologic signals. Circ..

[37] Rabiner L (1979). On the use of symmetry in FFT computation. *IEEE Transactions on Acoust*. Speech, Signal Process..

[38] Mahmoodabadi, S., Ahmadian, A. & Abolhasani, M. ECG feature extraction using daubechies wavelets. In *Proceedings of the fifth International conference on Visualization, Imaging and Image Processing (IASTED)*, 343–348 (2005). 10.1109/IEMBS.2005.1615314 17281084

[39] Němcová Andrea, Smíšek Radovan, Maršánová Lucie, Smital Lukáš, Vítek Martin (2018). A Comparative Analysis of Methods for Evaluation of ECG Signal Quality after Compression. BioMed Research International.

[40] Versen, M., Kipfelsberger, S. & Soekmen, F. Model-based reference design projects with mathworks’ HDL workflow advisor for custom-specific electronics with the Zedboard. In *ITG-Fachbericht 266: ANALOG 2016*, 1–4 (2016).

[41] Twomey, N. *et al*. The effect of lossy ECG compression on QRS and HRV feature extraction. In *2010 Annual International Conference of the IEEE Engineering in Medicine and Biology*, 634–637, 10.1109/IEMBS.2010.5627261 (IEEE, 2010). 10.1109/IEMBS.2010.5627261 21096542

[42] Zigel Y, Cohen A, Katz A (2000). The weighted diagnostic distortion (WDD) measure for ECG signal compression. IEEE transactions on biomedical engineering.

[43] Blanco-Velasco M, Cruz-Roldan F, Godino-Llorente J, Barner K (2004). ECG compression with retrieved quality guaranteed. Electron. Lett..

[44] Kwon O (2018). Electrocardiogram sampling frequency range acceptable for heart rate variability analysis. Healthc. Informatics Res..

[45] Lee S, Kim J, Lee M (2011). A real-time ECG data compression and transmission algorithm for an e-health device. IEEE Transactions on Biomed. Eng..

[46] Taddei A (1992). The european ST-T database: standard for evaluating systems for the analysis of ST-T changes in ambulatory electrocardiography. Eur. heart journal.

[47] Moody GB, Mark RG (2001). The impact of the MIT-BIH arrhythmia database. IEEE Eng. Medicine Biol. Mag..

[48] Behravan, V., Glover, N. E., Farry, R., Chiang, P. Y. & Shoaib, M. Rate-adaptive compressed-sensing and sparsity variance of biomedical signals. In *2015 IEEE 12th International Conference on Wearable and Implantable Body Sensor Networks (BSN)*, 1–6, 10.1109/BSN.2015.7299419 (IEEE, 2015).

[49] Association for the Advancement of Medical Instrumentation. Cardiac monitors, heart rate meters, and alarms. *Am. Natl. Standard (ANSI/AAMI EC13: 2002) Arlington, VA* 1–87, 10.13026/C2WC7M (2002).

[50] Johannesen L (2016). Late sodium current block for drug-induced long qt syndrome: results from a prospective clinical trial. Clin. Pharmacol. & Ther..

[51] Adamo A, Grossi G, Lanzarotti R, Lin J (2015). ECG compression retaining the best natural basis k-coefficients via sparse decomposition. Biomed. Signal Process. Control..

[52] Lai, S.-C. *et al*. An efficient DCT-IV-based ECG compression algorithm and its hardware accelerator design. In *2013 IEEE International Symposium on Circuits and Systems (ISCAS2013)*, 1296–1299, 10.1109/ISCAS.2013.6572091 (IEEE, 2013).

[53] Luo C-H (2016). An ECG acquisition system prototype design with flexible PDMS dry electrodes and variable transform length DCT-IV based compression algorithm. IEEE Sensors J..

