# Supplementary figures and images for: Reconfigurable Architecture for Multi-lead ECG Signal Compression with High-frequency Noise Reduction
Source: Sci Rep. 2019 Nov 21;9:17233. doi: 10.1038/s41598-019-53460-3 (PMC6872821; doi:10.1038/s41598-019-53460-3)

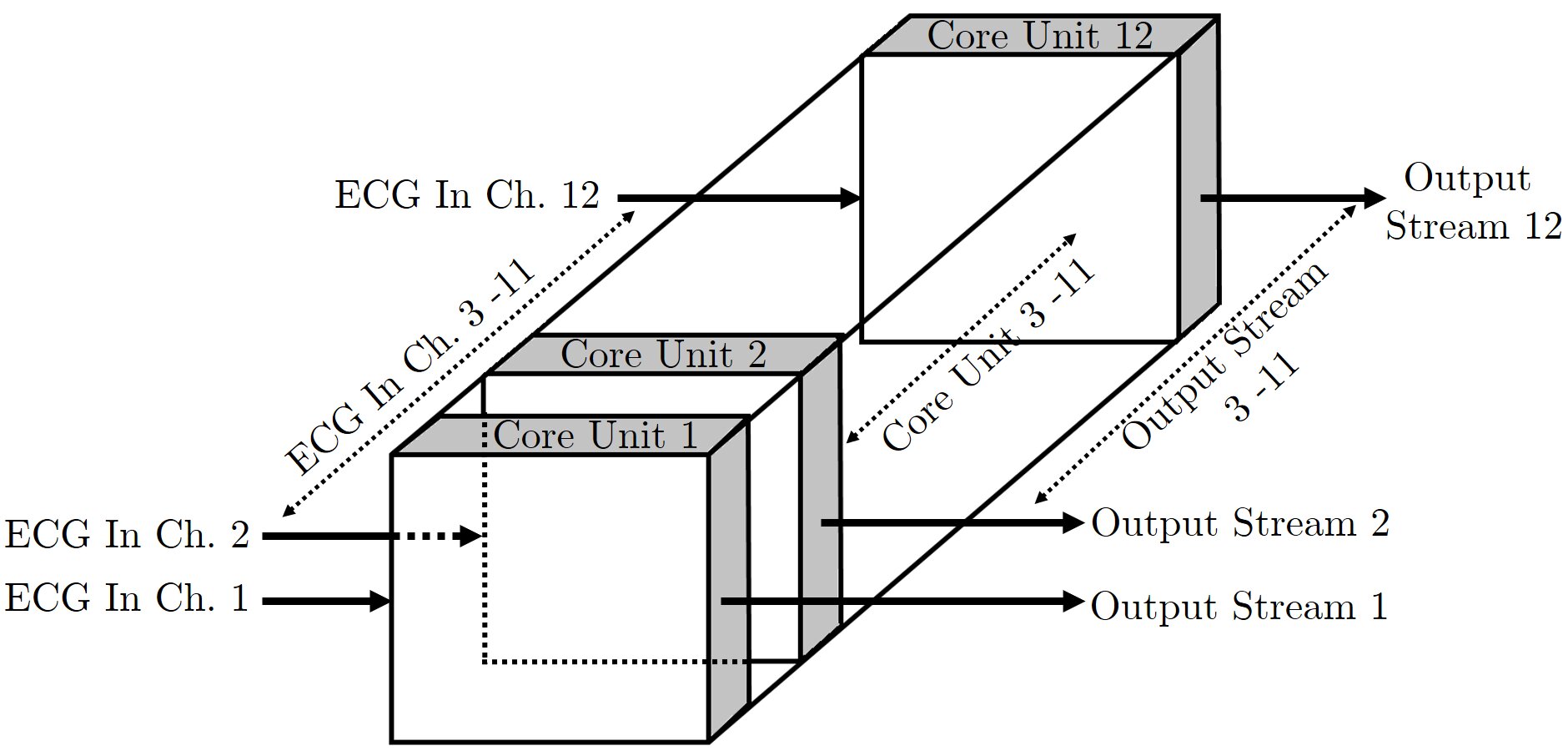

Supplement: Supplementary file 1 — LaTeX Supplementary File [file 41598_2019_53460_MOESM1_ESM.zip › Arch_1.jpg]

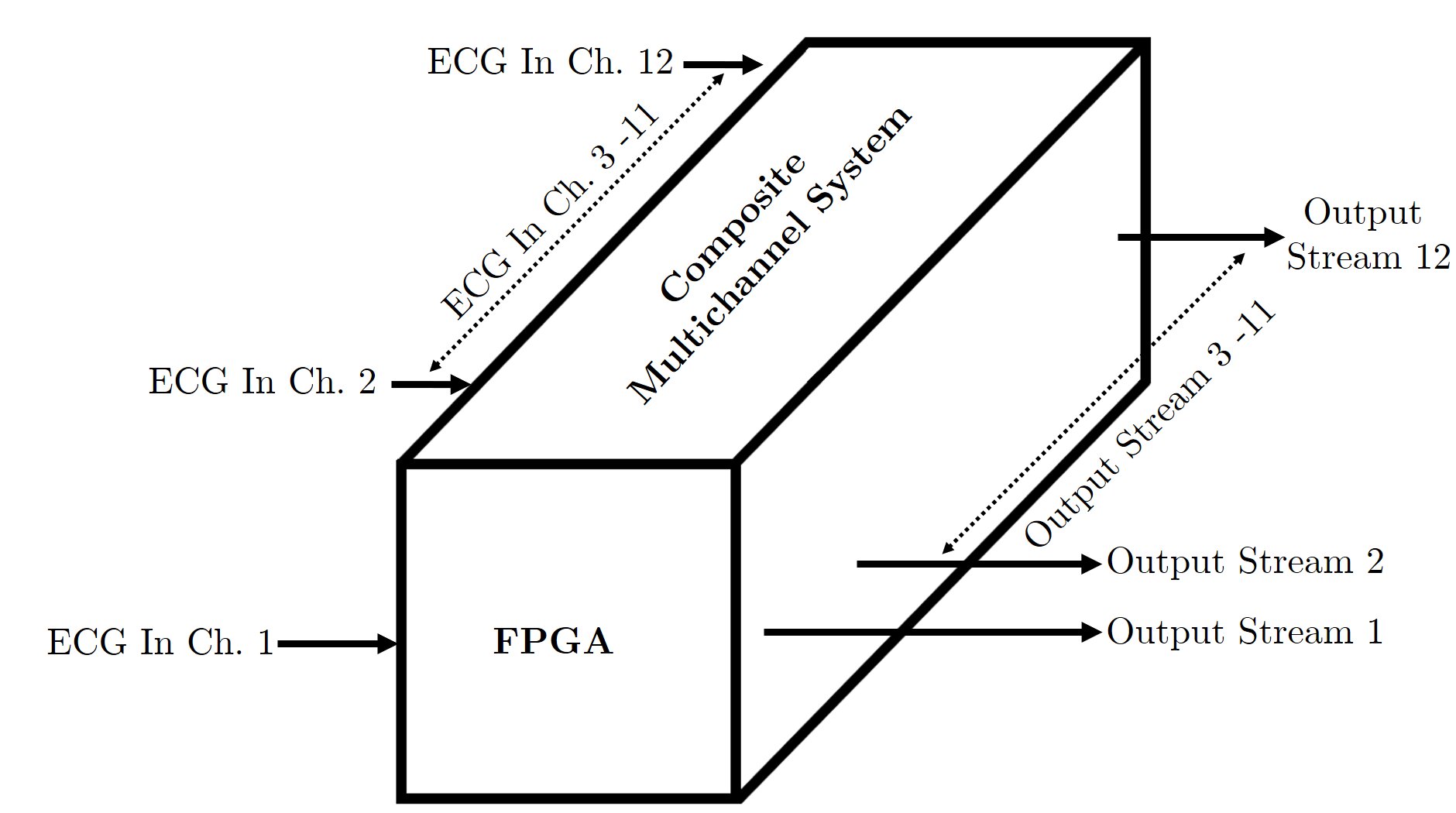

Supplement: Supplementary file 1 — LaTeX Supplementary File [file 41598_2019_53460_MOESM1_ESM.zip › Arch_2.jpg]

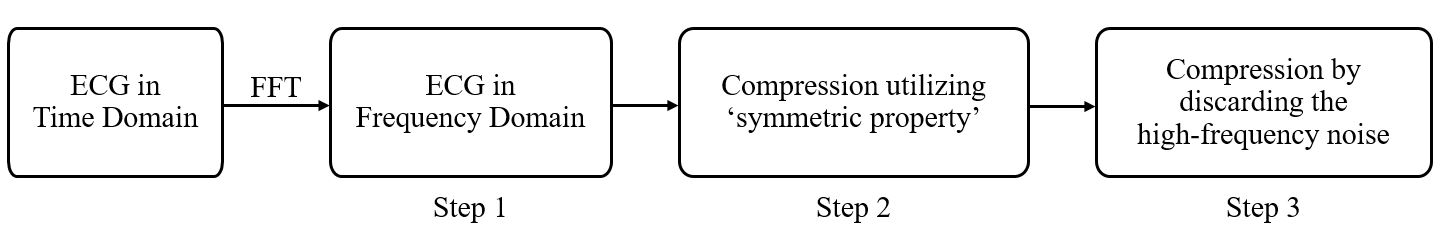

Supplement: Supplementary file 1 — LaTeX Supplementary File [file 41598_2019_53460_MOESM1_ESM.zip › block_dg_1.JPG]

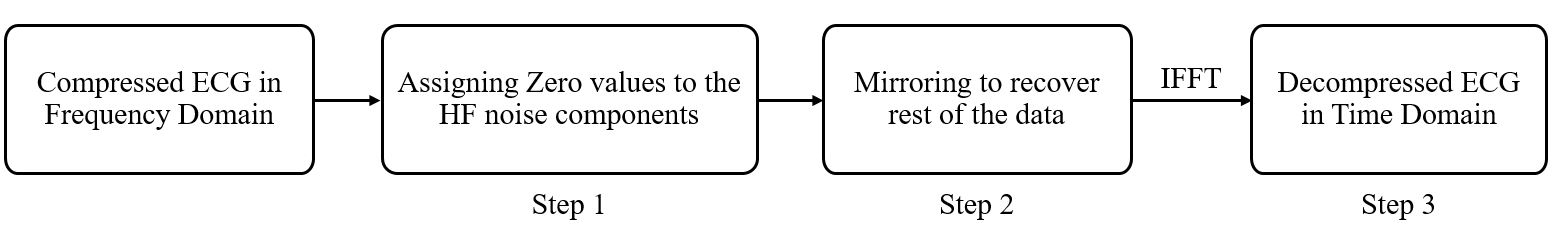

Supplement: Supplementary file 1 — LaTeX Supplementary File [file 41598_2019_53460_MOESM1_ESM.zip › block_dg_2.JPG]

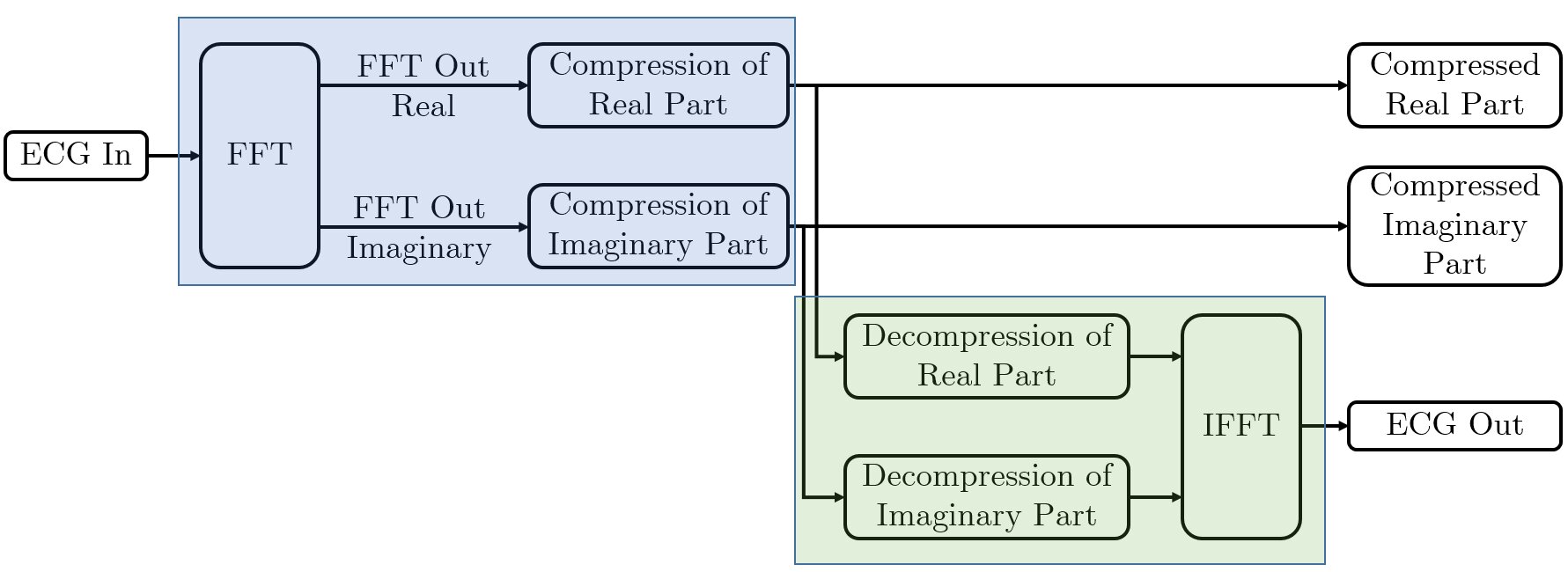

Supplement: Supplementary file 1 — LaTeX Supplementary File [file 41598_2019_53460_MOESM1_ESM.zip › block_dg_3.jpg]

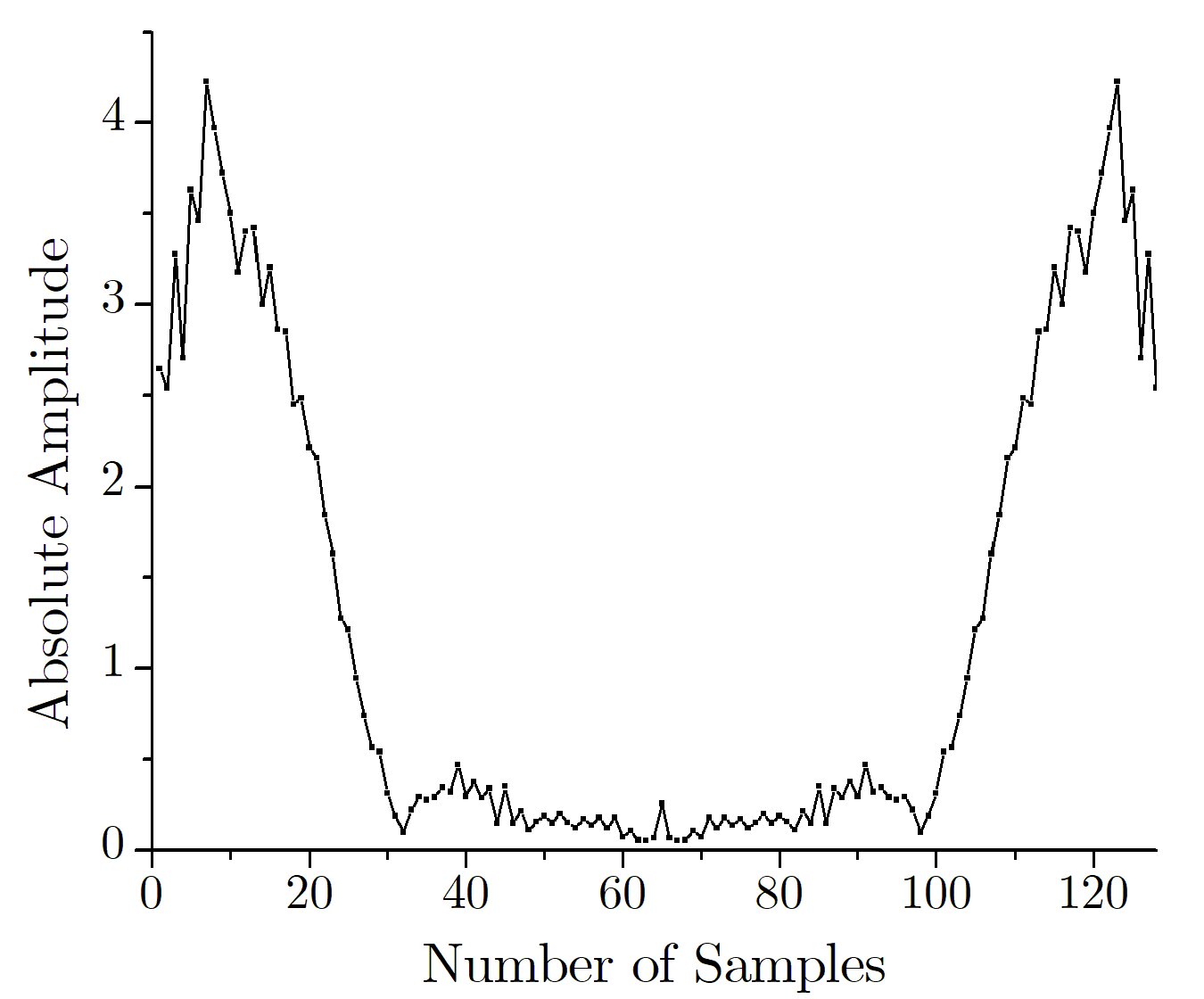

Supplement: Supplementary file 1 — LaTeX Supplementary File [file 41598_2019_53460_MOESM1_ESM.zip › ECG_FFT.jpg]

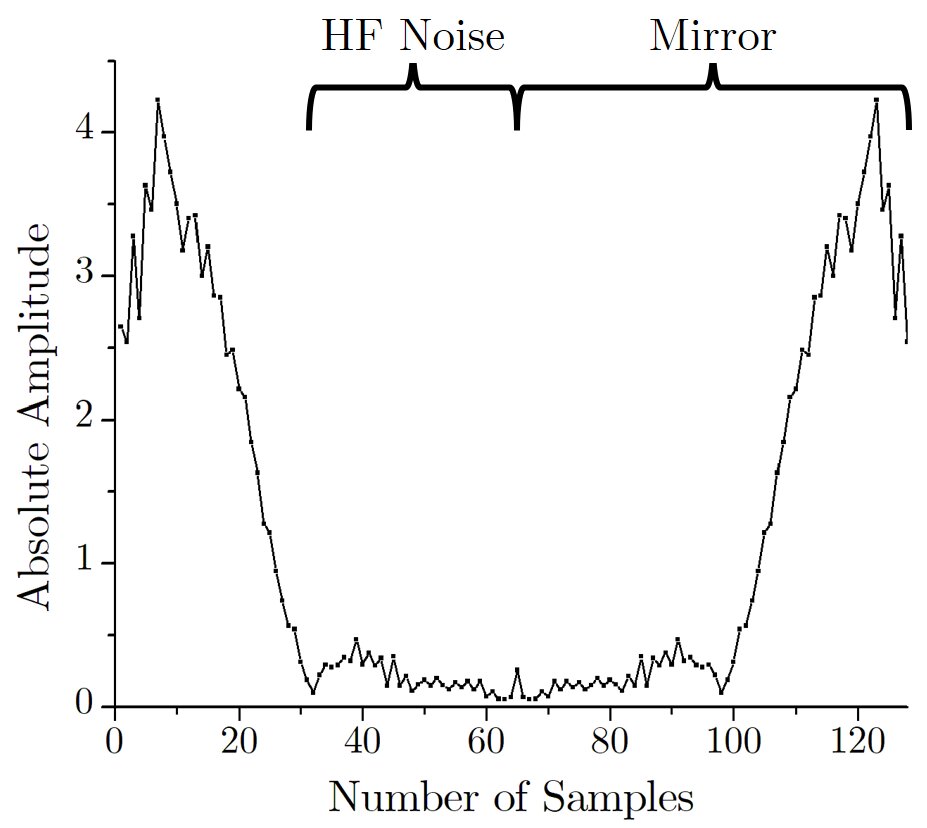

Supplement: Supplementary file 1 — LaTeX Supplementary File [file 41598_2019_53460_MOESM1_ESM.zip › ECG_FFT_2.jpg]

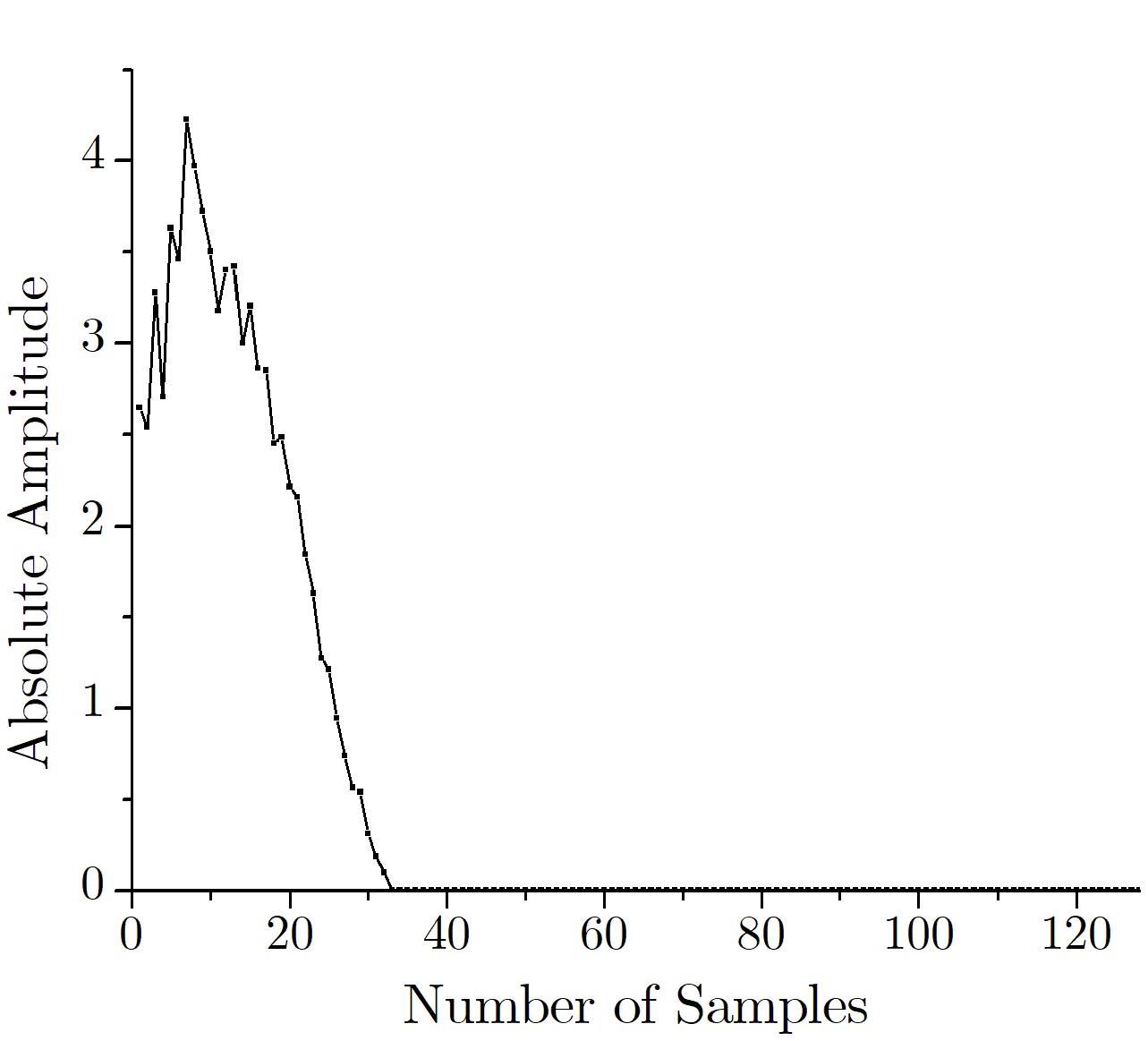

Supplement: Supplementary file 1 — LaTeX Supplementary File [file 41598_2019_53460_MOESM1_ESM.zip › ECG_FFT_Comp.jpg]

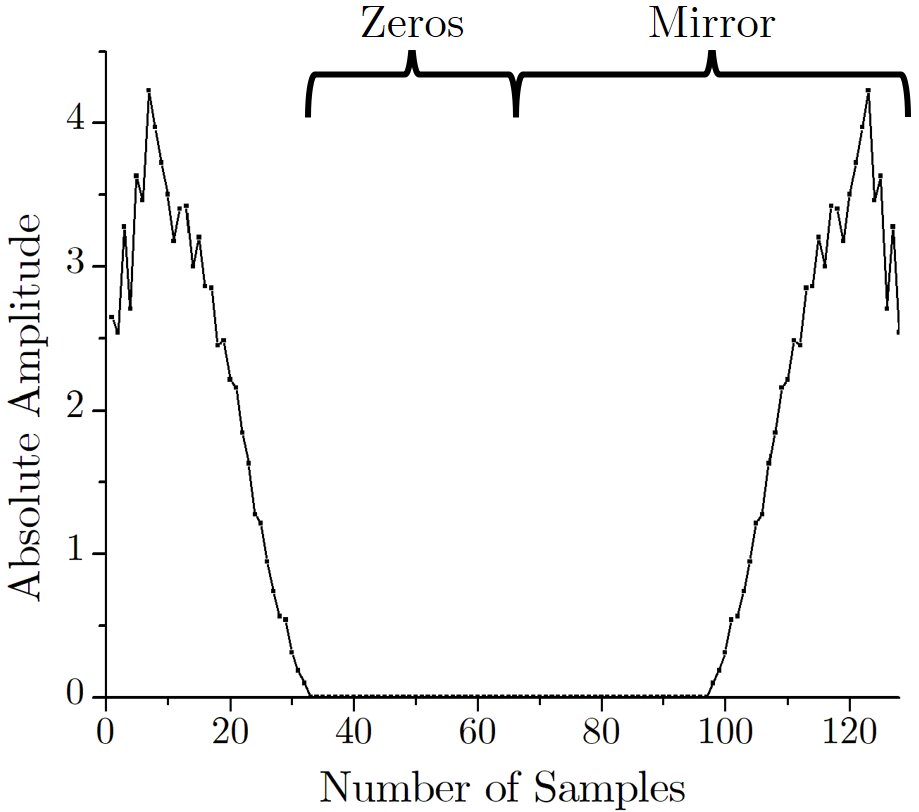

Supplement: Supplementary file 1 — LaTeX Supplementary File [file 41598_2019_53460_MOESM1_ESM.zip › ECG_FFT_DeComp.jpg]

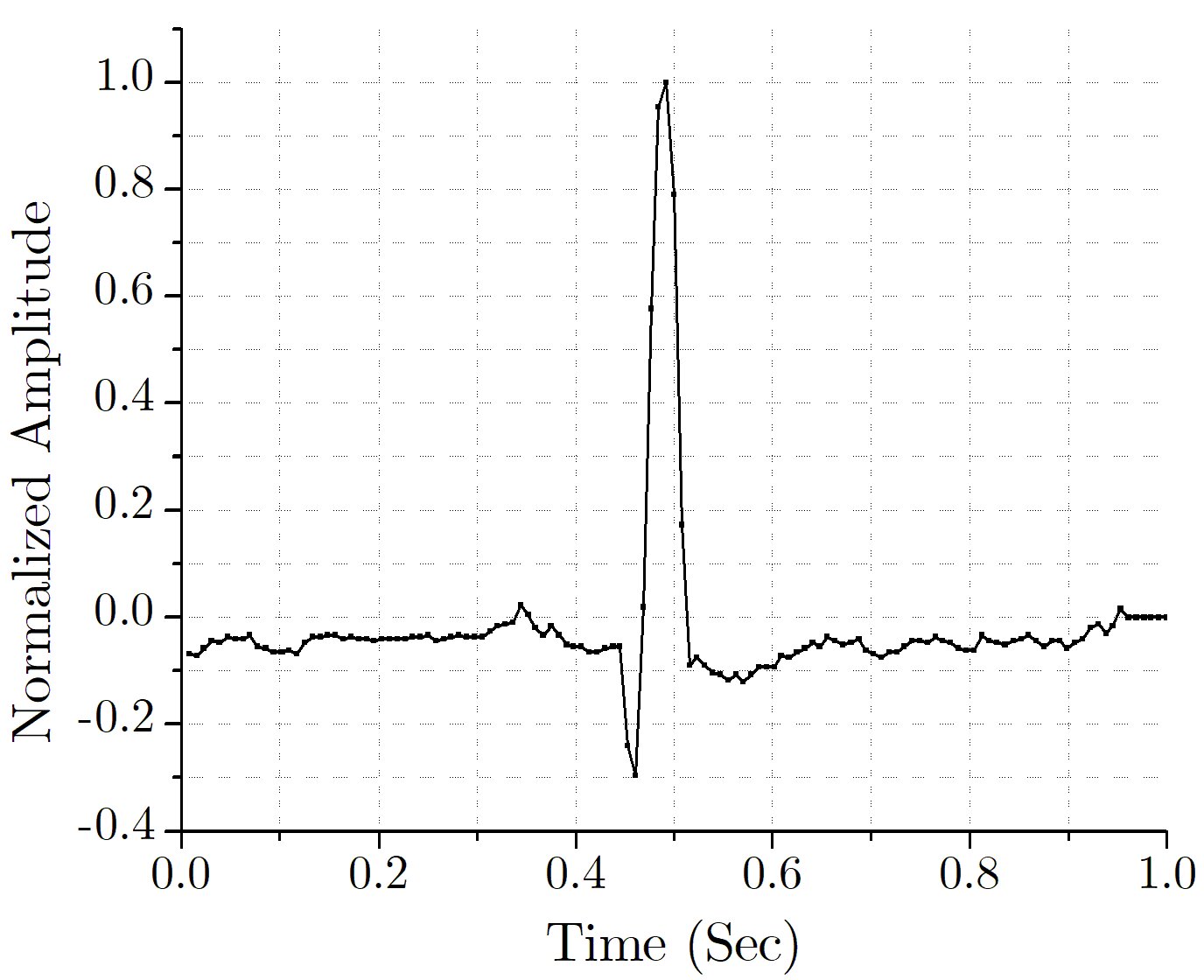

Supplement: Supplementary file 1 — LaTeX Supplementary File [file 41598_2019_53460_MOESM1_ESM.zip › ECG_IN.jpg]

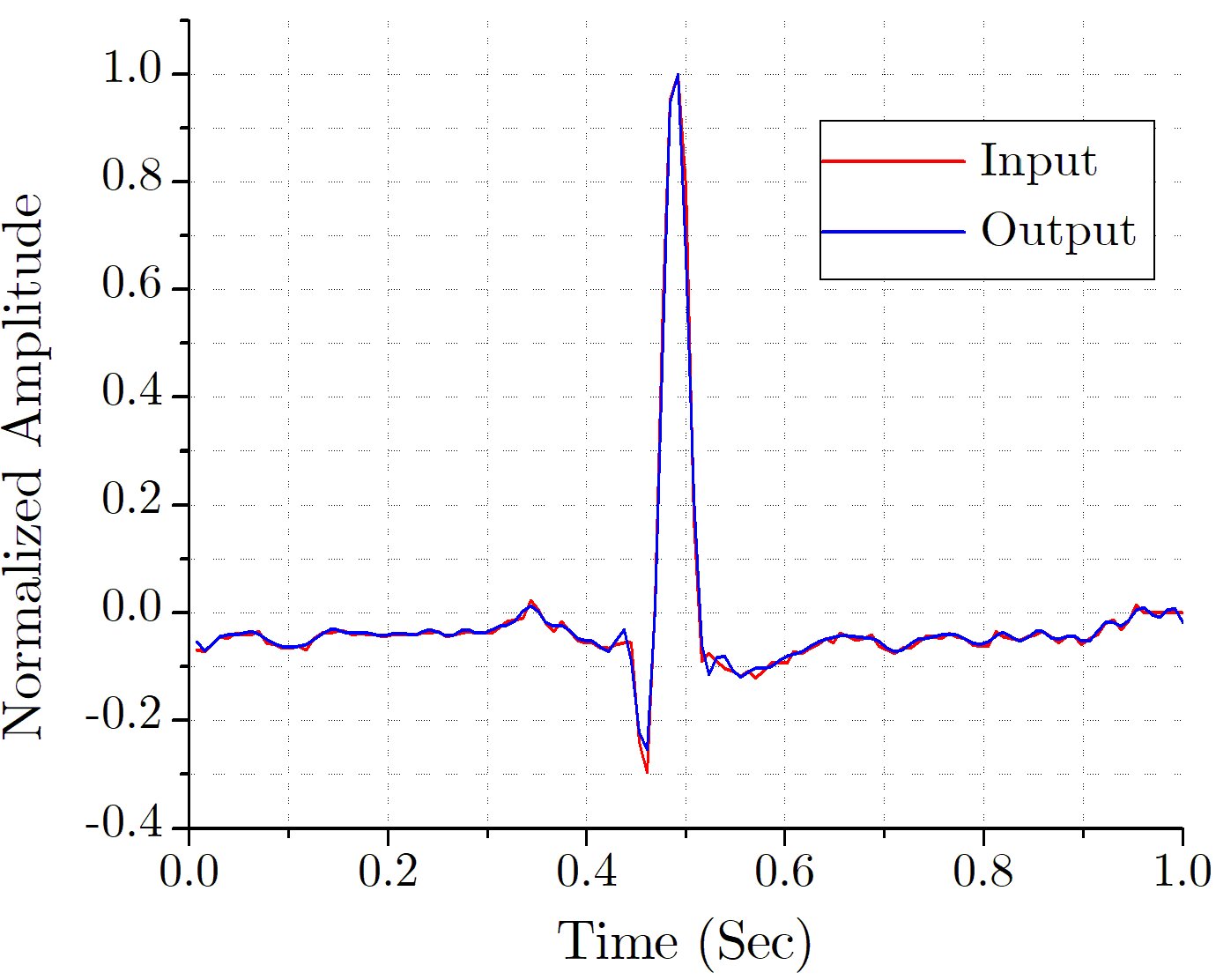

Supplement: Supplementary file 1 — LaTeX Supplementary File [file 41598_2019_53460_MOESM1_ESM.zip › ECG_IO_Compare.jpg]

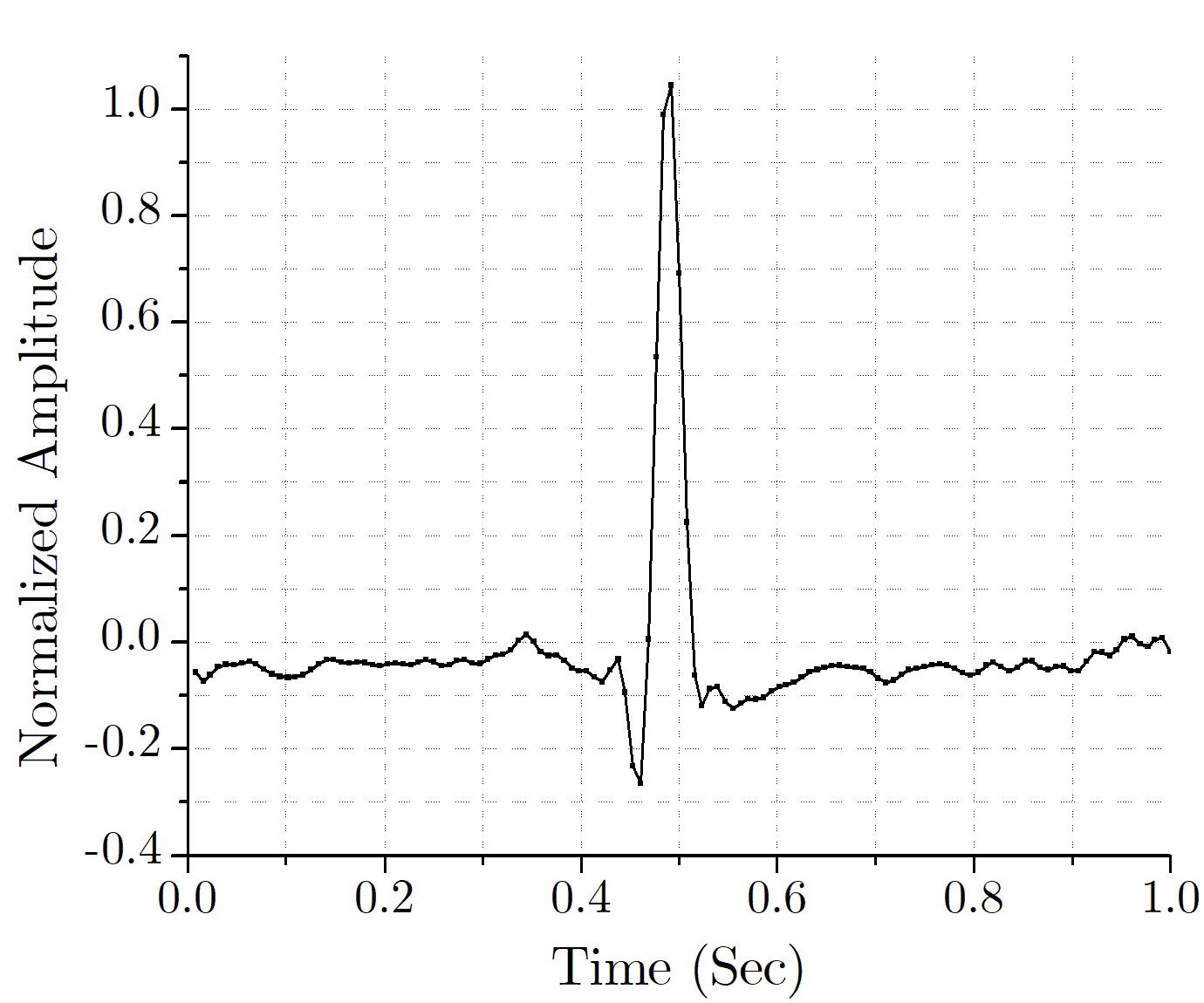

Supplement: Supplementary file 1 — LaTeX Supplementary File [file 41598_2019_53460_MOESM1_ESM.zip › ECG_Out.jpg]

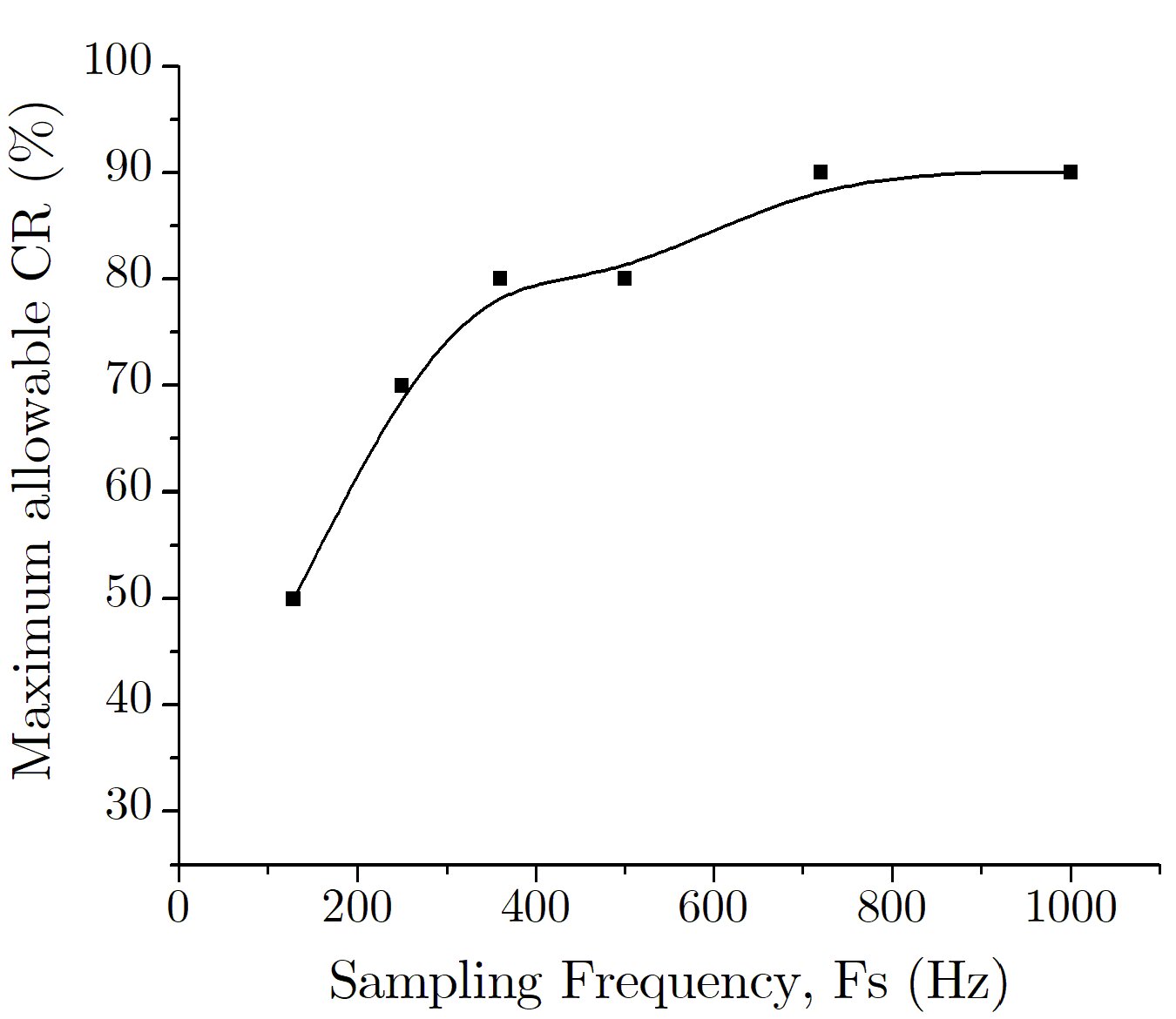

Supplement: Supplementary file 1 — LaTeX Supplementary File [file 41598_2019_53460_MOESM1_ESM.zip › Max_CR.jpg]

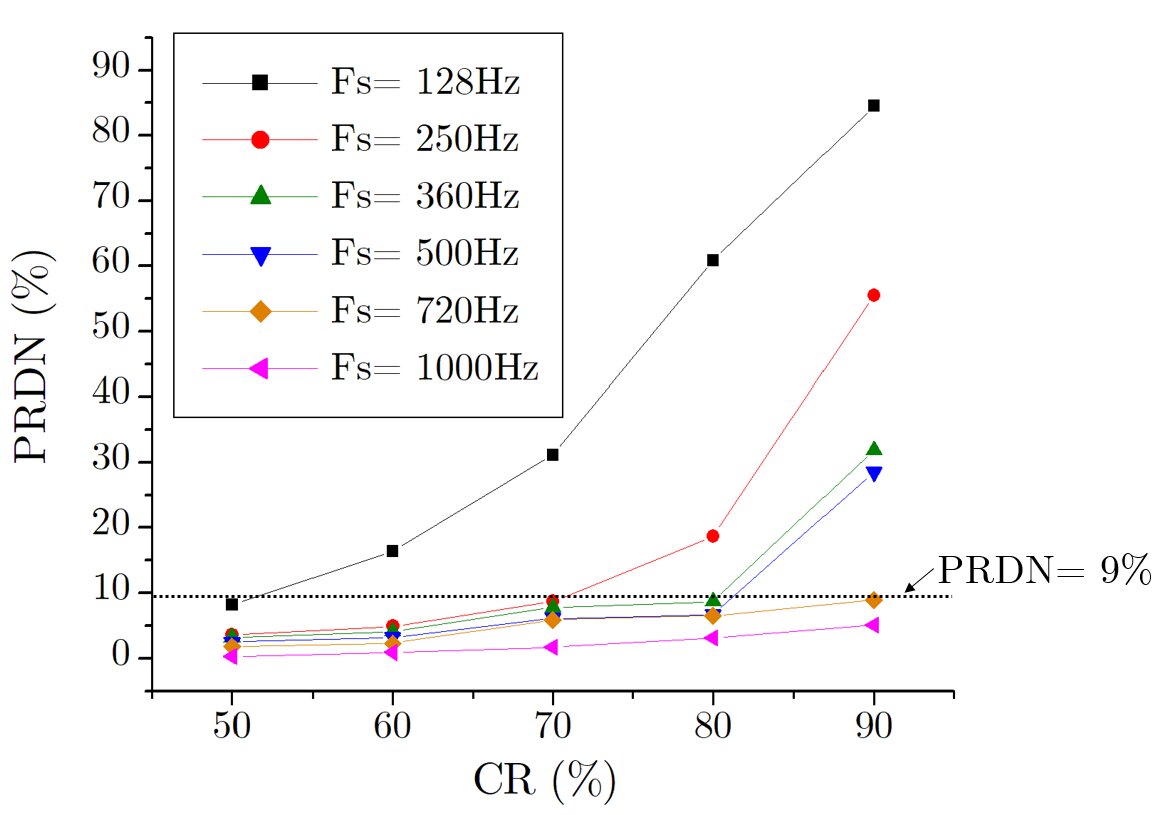

Supplement: Supplementary file 1 — LaTeX Supplementary File [file 41598_2019_53460_MOESM1_ESM.zip › Result1.jpg]

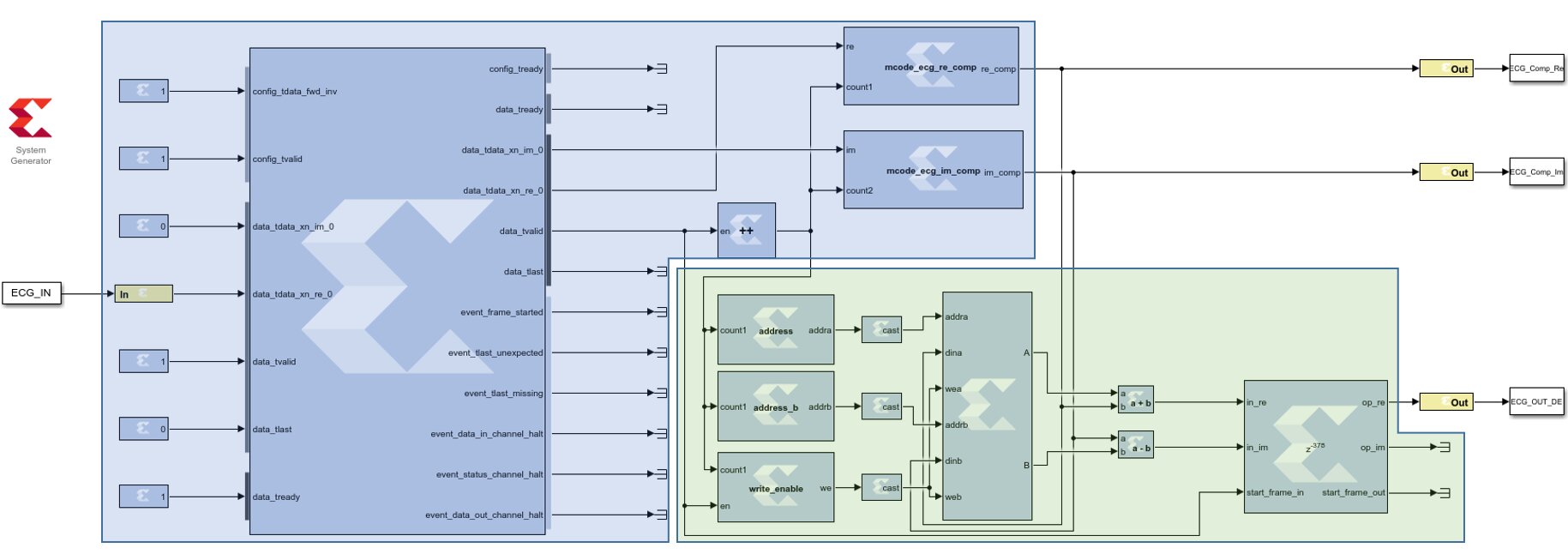

Supplement: Supplementary file 1 — LaTeX Supplementary File [file 41598_2019_53460_MOESM1_ESM.zip › SysGen.jpg]

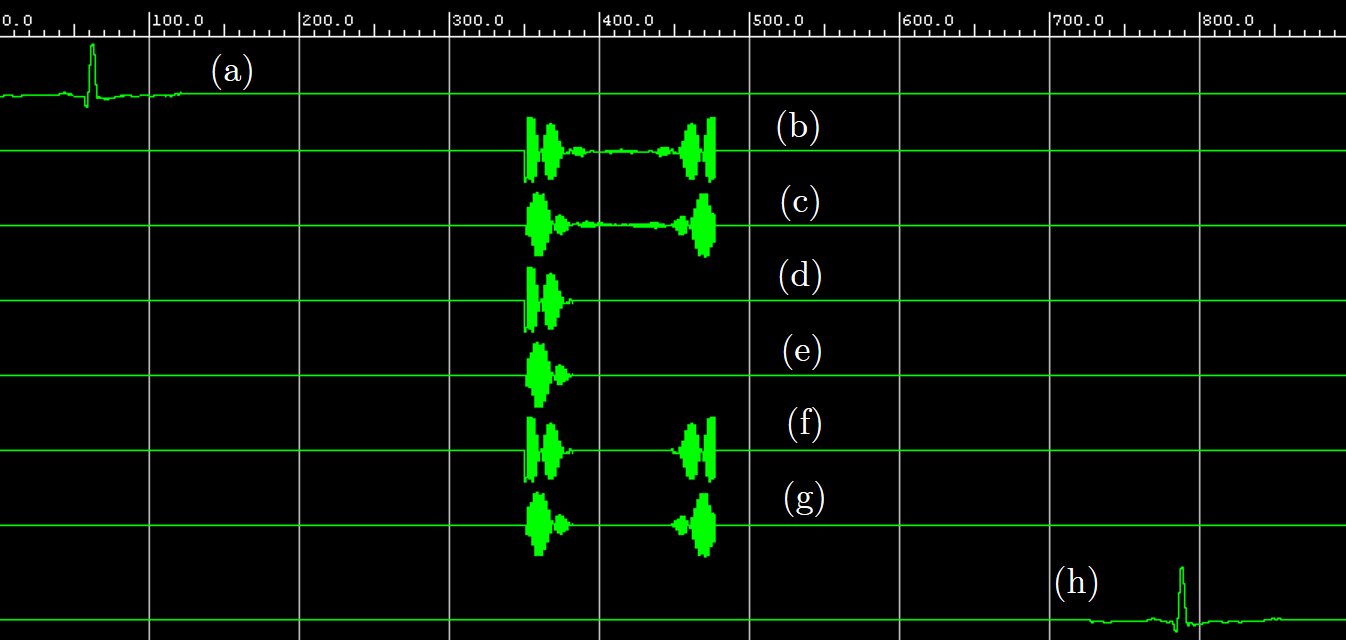

Supplement: Supplementary file 1 — LaTeX Supplementary File [file 41598_2019_53460_MOESM1_ESM.zip › SysGen_op_i.jpg]
